# Flaying Criticism

**Published:** 1844-08-24

**Authors:** 


FLAYING CRITICISM.

We extract the following *notice* from the last number of the *Medico-Chirurgical Review.*

VIVISECTION.—LETTER TO THE EARL OF CAERNARVON. FROM RICHARD JAMESON, ESQ.

It appears that Lord Caernarvon is President of the "*Society for Preventing Cruelty to Animals*," and, as such, has been holding forth against the high crimes and misdemeanors of the medical profession for torturing animals to death in their physiological experiments! A certain Rev. J. Styles has gained a prize of £100 for the best Essay on this Pseudo-philanthropy, and has informed the public—"that every surgeon's apprentice thinks himself entitled to find his way into the arcana of Nature by scalping cats and rabbits *to see where their brains lie.*" This is not merely a "*clerical* error," as the lawyers say, but a clerical lie. Admitting, for the sake of argument, that the medical profession should exhibit, among its numerous ranks, an instance or two of such senseless cruelty, is that any reason for using the sweeping assertion that "*every surgeon's apprentice, &c. &c.*" Does the ecclesiastical world never exhibit a black sheep?—If, because a Bishop was seen running down St. James's street with his "*tights*" about his heels, we were to assert that every clergyman was a ———, what would this mendacious John Styles say to the accusation?

The only apology that can be offered for this Doctor of Divinity is, that he is a credulous ass. Thus, to astonish the Gobe-Mouches among the auditors, he tells them that oxen are driven many days without food, with "their hoofs worn off, and on *bleeding stumps!!*" Why Baron Munchausen was nothing to this D. D.! Can he be so silly too as to suppose that a grazier or a butcher would starve his cattle for days together, and thus to reduce their weight and condition as to suffer a considerable loss in the sale? This sapient philosopher tells us that the Hippopotamus, whose side would resist a musket bullet, has recourse to phlebotomy, when sick, by running against the point of a reed!! By way of contrast to the surgeons' apprentices, he informs us that predaceous animals destroy their victims with the least possible amount of pain, and that it is usually in the night time, when they are asleep, that they pounce on their prey, murdering them before they have time to reflect on their own danger! What tender-hearted creatures these tigers, hyenas, cats, and owls are! It is not because they can surprize their prey more easily in the dark than in the light, that they prowl about after sunset, but entirely to save the deer, goats, mice, and sparrows, the pain of being slaughtered! See how the cat domesticated among our amiable tabbies, fondles and soothes its victim the mouse, before it gives the final coup de grace!

But look at MAN himself. Leaving aside the wars, murders and persecutions of all ages, see how many thousands of animals are annually slaughtered in the most cruel manner, by your nobility and gentry, while hunting the stag, the hare, &c. and that for mere sport, and without any regard to science or ultimate utility. The subject is extremely well handled by Mr. Jameson, and does great credit to his head and his heart. We strongly recommend its perusal.

